# SOCIAL CONNECTIONS AND HEALTH AMONG OLDER ADULTS IN SENIOR HOUSING COMMUNITIES

**DOI:** 10.1093/geroni/igz038.2164

**Published:** 2019-11-08

**Authors:** Harry O Taylor, Nancy Morrow-Howell

**Affiliations:** 1 Washington University in St. Louis, St. Louis, Missouri, United States; 2 Brown School, Washington University in St. Louis, St. Louis, Missouri, United States

## Abstract

Our study describes social connections among residents in two low-income senior housing communities and then examines if these connections influence their well-being. Operationalization of social connections included social network size, informal social support, social engagement, and loneliness. The Convoy Model of Social Relations guides our study in identifying objective and subjective social connections and examining how they affect well-being. Most residents maintained active connections: 53% saw five or more family members in the previous month, 52% felt they could rely on their family members and over 60% participated in group and/or Church activities; however, 70% of residents were moderately or severely lonely. Greater loneliness and less informal social support were associated with worse self-rated physical health, worse mental health, and lower life satisfaction. This residential setting offers promise for developing interventions to decrease loneliness and strengthen social connections to improve residents’ health and well-being.

